# A Trespasser in Lymph Node: A Case Report

**DOI:** 10.7759/cureus.69266

**Published:** 2024-09-12

**Authors:** Priyankha Ramamoorthy, Barathi Gunabooshanam, Subalakshmi Balasubramanian

**Affiliations:** 1 Pathology, Sri Ramachandra Institute of Higher Education and Research, Chennai, IND

**Keywords:** cerebrospinal fluid (csf), cryptococcus neoformans, fine needle aspiration cytology, lymphadenitis, lymph node

## Abstract

Cryptococcal meningitis is a prevalent, opportunistic fungal disease seen in human immunodeficiency virus (HIV)-infected individuals. A lymph node is an unusual presentation site for *Cryptococcus* and can mimic tuberculosis. Disseminated cryptococcosis is a life-threatening disease that is seen commonly in acquired immunodeficiency syndrome (AIDS).

We report a case of an HIV patient who presented with mild pleural effusion, multiple mediastinal, axillary lymphadenopathy with a low CD4:CD8 lymphocyte ratio, and favored clinically disseminated tuberculosis. Further cerebrospinal fluid (CSF) and tracheal aspirate have been done. Tracheal aspirate culture shows a fungal organism resembling *Cryptococcus*. Later, India ink staining on CSF highlighted the fungal organism *Cryptococcus*. Cytopathological investigation showed necrotizing inflammation along with fungal organisms, confirming the presence of cryptococcal lymphadenitis.

## Introduction

Cryptococcosis is a common opportunistic infection caused by *Cryptococcus neoformans*, usually in patients with acquired immunodeficiency syndrome (AIDS), which is widely disseminated and life-threatening [1]. There has been a dramatic increase in patients in the ensuing years with AIDS or other states of immune compromise. Globally, cryptococcal disease accounts for 19% of AIDS-related mortality by Rajasingham et al.’s study [2]. The infection commences in the respiratory tract but also involves other systems in our body, like the central nervous system (CNS), lungs, skin, lymph nodes, bone marrow, gastrointestinal tract, retina, liver, and spleen. Cryptococcal infections usually manifest as meningoencephalitis, followed by pulmonary and skin diseases. However, lymph node involvement is uncommon in cryptococcosis; when it does occur, it is often referred to as “lymphonodular cryptococcosis,” which is more frequently reported in children [3]. 

In patients with cryptococcal lymphadenitis, fine needle aspiration cytology (FNAC) of lymph nodes provides an economical and quicker accomplished cyst diagnosis.

## Case presentation

A 42-year-old male patient is admitted with symptoms of fever and breathlessness. He is a known case of human immunodeficiency virus (HIV) infection and is on irregular treatment. On examination, the patient is found to have multiple lymphadenopathy. His CD4:CD8 lymphocyte ratio is 0.06. His radiology reveals consolidation of both lungs, multiple mediastinal and bilateral axillary lymphadenopathy, multiple scattered nodules, mild pleural effusion, and some areas of consolidation owing to the possibility of disseminated tuberculosis. His blood investigations show low hemoglobin with an increase in total blood count, erythrocyte sedimentation rate (ESR), and elevated renal profile (Tables 1, 2).

**Table 1:** Blood investigations

S. no	Test	Result	Normal values
1	Total leulocyte count	16,700 cells/cu.mm	4000-11,000 cells/cu.mm
2	Erythrocyte sedimentation rate (ESR)	78 mm	Adult male: <15 mm/h; adult female: <20 mm/h
3	Hemoglobin	9 g/dL	13-17 g/dL

**Table 2:** Biochemical investigations

S. no	Test	Result	Normal values
1	Blood urea nitrogen	52 mg/dL	7-18 mg/dL
2	Serum creatinine	1.8 mg/dL	0.90-1.30 mg/dL
3	Serum potassium	6.0 mmol/L	3.5-5.0 mg/dL

**Figure 1:**
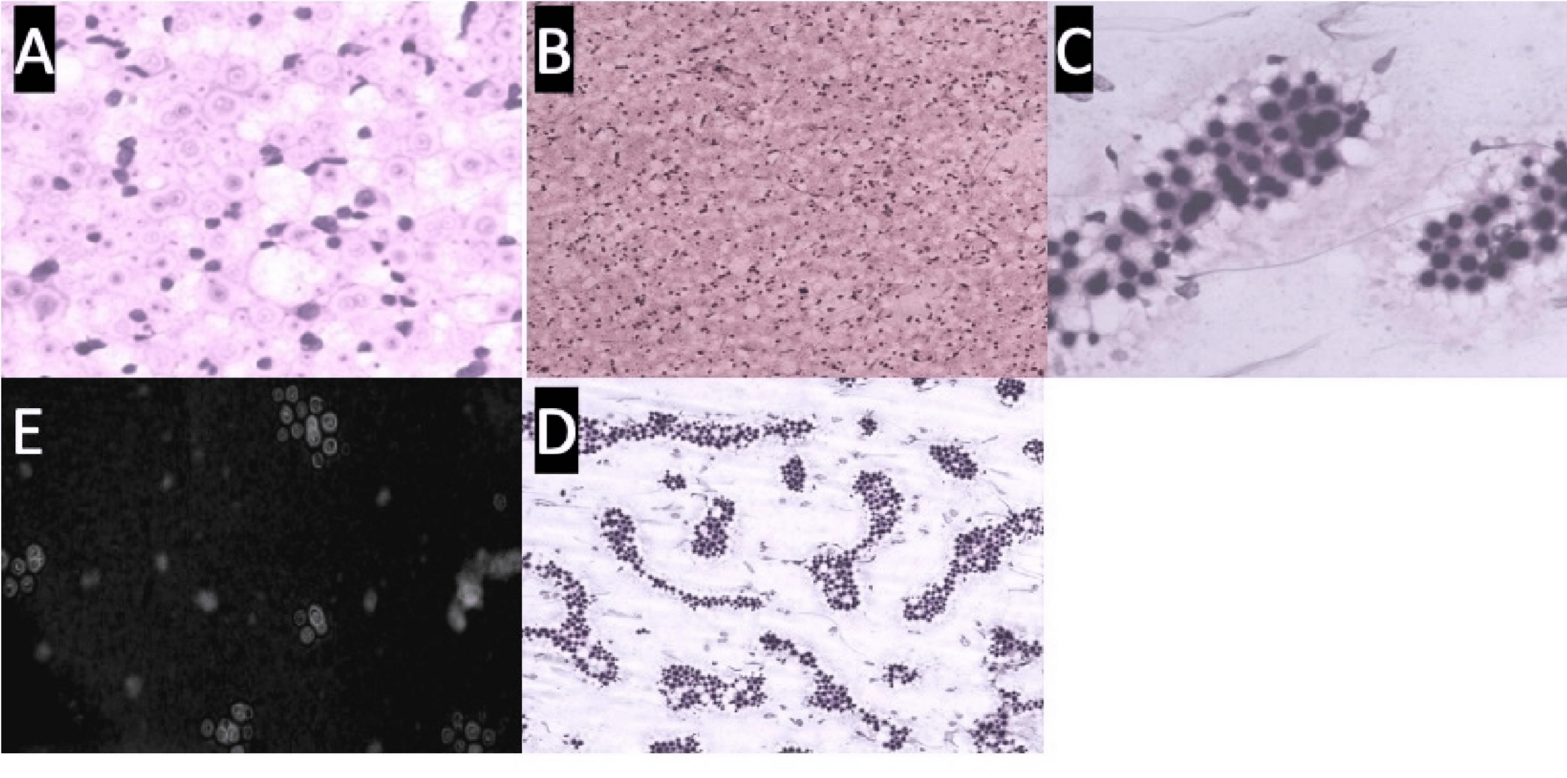
Microscopic images (A) 40×, H&E, microscopic images showing several round encapsulated microorganisms with a “halo” appearance resembling *Cryptococcus*. (B) 10×, H&E, necrotizing inflammation with organisms resembling *Cryptococcus*. (C,D) 40× and 10×, PAS, microscopic images of cryptococcal organisms in a lymph node, respectively. (E) Light microscopic image of India ink staining: *C. neoformans* highlighting the capsule around the organism, making it visible as a halo.

On further examination, a palpable cervical lymph node measuring 1.5 × 1 cm, along with the axillary lymph node, is found. Cerebrospinal fluid (CSF) is sent for cytology in view of disseminated tuberculosis, followed by tracheal aspirate culture. FNAC from the lymph node shows necrotizing lymphadenitis admixed with fungal organisms resembling *Cryptococcus*, which is confirmed by special stains. Further CSF analysis and tracheal aspirate also show fungal elements (Tables 3-5). India ink staining from the CSF highlights the budding yeast form of *Cryptococcus* with its thick, gelatinous capsule (Figure 1).

**Table 3:** CSF analysis

S. no	Test	Result	Normal values
1	CSF protein	57	15-45 mg/dL
2	CSF cell count	2.5 mL of fluid received; WBC: nil; RBC: nil	Nil
3	CSF xanthochromia	Absent	Nil

**Table 4:** Microbiological analysis of AFB/Gram staining

S. no	Microscopy
1	Direct smear AFB: acid-fast bacilli not seen in smear
2	Direct smear Gram stain: few pus cells and moderate budding yeast cells seen
3	India ink preparation + cryptococcal antigen: positive for capsulated budding yeast cells; latex agglutination test: positive Gram stain, occasional inflammatory cells, and occasional budding yeast cells

**Table 5:** Culture report

S. no	Nature of specimen	Microscopy	Result
1	Culture of blood	Cryptococcus neoformans	Cryptococcus neoformans
2	Culture of CSF	Moderate number of polymorphs and few budding yeast cells	Cryptococcus neoformans
3	Culture of tracheal aspirate	Moderate pus cells and few budding yeasts seen	Cryptococcus neoformans
4	Culture of urine	Few pus cells and few Gram-positive cocci in chains	Enterococcus faecalis

Correlating the radiology, cytology, and various laboratory investigations favors the presence of fungal organisms resembling *Cryptococcus*. Thus, cryptococcal lymphadenitis is rendered. He is treated with antibiotics for bronchopneumonia and has been started on fluconazole after the diagnosis was made.

His radiological investigations supported the diagnosis of bronchopneumonia, secondary to disseminated tuberculosis/opportunistic infection, and his culture also aided in the diagnosis. The final diagnosis of cryptococcal bronchopneumonia with lymphadenitis was made. He was treated with antibiotics for bronchopneumonia and started on fluconazole after the diagnosis was made.

Despite rapid diagnosis, our patient succumbed to the disease.

## Discussion

*Cryptococcus* enters the body through inhaled spores or dried yeast, affecting the lungs. However, the fungus often targets the central nervous system (CNS), causing potentially fatal meningitis.

Studies in India reveal a prevalence of *Cryptococcus* infection among HIV-positive individuals, ranging from 1.5% to 10%. An uncommon but concerning form of extrapulmonary cryptococcosis is lymphadenitis, involving the lymph nodes. This manifestation is even recognized as an AIDS-defining criterion by the Centre for Disease Control and Prevention (CDC) [4-6]. *Cryptococcus* can hide in various parts of the body, as well as in fluids like spinal fluid (CSF) and mucus from coughs (sputum) or bronchoscopies. Needle biopsies (FNAC) of lymph nodes, organs like the thyroid and spleen, and even bones and lungs can reveal its presence [7-9]. Under the microscope, *Cryptococcus* is identified by thin-walled, dark-colored cells with a distinct, clear halo around them. Special stains, like PAS, can help highlight these invaders for easier detection [10]. The organism is surrounded by a mucopolysaccharide capsule measuring 5-15 μm in diameter. Special stains (Gomori’s methenamine silver, PAS, and mucicarmine) aid in identifying this organism. India ink and PAS staining done in our FNAC smear highlight the budding yeast and capsule forms. Commonly, intravenous amphotericin B and oral fluconazole are given as treatment. However, oral flucytosine can also be given [11].

An expeditious diagnosis is of very vital because dissemination of cryptococcal infection becomes life-threatening. Pulmonary, intestinal, bone marrow, and retinal involvement have also been described. Diagnosis of cryptococcal lymphadenitis remains a potential challenge as it can be subclinical with a lack of characteristic clinical and radiological features. 

Although many cases of cryptococcal infection worldwide have been reported in other organs, it is sporadic to diagnose cryptococcal infection in lymph nodes by FNAC.

## Conclusions

The minimally invasive nature of FNAC, coupled with its ability to provide quick results and guide treatment decisions through smears and cultures, makes it a patient-friendly and efficient addition to routine diagnostic practices. Lymph-node FNAC represents a first-line diagnostic option for immunocompromised patients presenting with a wide range of potential diagnoses. This approach holds promise for definitive diagnoses and timely treatment initiation. The definitive diagnosis of this case of cryptococcosis was possible only because of the simple, cost-effective technique of FNAC, which prompted the early initiation of specific and life-saving treatment.
